# Morpho-Functional Responsiveness of Caco-2 Enterocyte-like Monolayers to Insulin in a Pro-Inflammatory Environment

**DOI:** 10.3390/cells14171358

**Published:** 2025-08-31

**Authors:** Aurora Mazzei, Marina Damato, Ilenia Iaia, Michele Maffia, Roberta Schiavone, Tiziano Verri, Amilcare Barca

**Affiliations:** 1 Laboratory of Applied Physiology, Department of Biological and Environmental Sciences and Technologies, University of Salento, 73100 Lecce, Italy; aurora.mazzei@unisalento.it (A.M.); roberta.schiavone@unisalento.it (R.S.); tiziano.verri@unisalento.it (T.V.); 2 Laboratory of Human Physiology, Department of Experimental Medicine, University of Salento, 73100 Lecce, Italy; marina.damato@unisalento.it (M.D.); michele.maffia@unisalento.it (M.M.); 3 Institute of Clinical Physiology, National Research Council IFC-CNR, 73100 Lecce, Italy; ileniaiaia@cnr.it; 4 Laboratory of Applied Physiology, Department of Experimental Medicine, University of Salento, 73100 Lecce, Italy

**Keywords:** intestinal epithelium, Caco-2 monolayers, insulin signaling pathways, inflammation

## Abstract

In exerting its actions on the utilization and storage of nutrients, the hormonal effects of insulin (INS) on target cells include important changes in terms of cell morphology involving cytoskeletal actin. Sensitivity to INS affects intestinal epithelial cells, which express receptors through which tight junctions and barrier permeability are also modulated. Nevertheless, the impact of INS on physiological rather than pathophysiological processes along gastrointestinal epithelia is not fully established. Here, we investigate INS effects on differentiated Caco-2 monolayers challenged by inflammatory stimuli, i.e., interleukin 1 beta (IL-1β) and interferon gamma (IFN-γ), aiming to identify morpho-functional variations potentially associated with INS-dependent responses in intestinal epithelia differentially driven by different inflammation mediators. By observing the actin cytoskeleton, we characterized the impact of INS on actin structures’ organization, both in the absence and presence of pro-inflammatory treatments. Coherently, we observed altered expression of proteins interrelated to cytoskeletal dynamics (FAK, ITGB1), particularly evident in the synergistic action of IFN-γ and INS, also confirmed by the impact on INS-mediated regulation of the MAPK signalling pathway. Overall, the results describe a modular responsiveness of enterocyte-like monolayers to INS, depending on different inflammatory mediators, hinting at the interplay between INS signalling and morpho-functional remodelling in intestinal epithelial cells.

## 1. Introduction

Insulin (INS) is a hormone with pleiotropic effects, including a wide range of metabolic, nuclear, and morphological actions in various types of cells. Additionally, INS exerts specific effects on cellular morphology, e.g., as an inducer of rapid and marked rearrangement of the actin cytoskeleton, which in turn facilitates further propagation of the effects of the hormone itself [1].

At the molecular level, INS binds to its membrane receptor (INSR), activating intrinsic tyrosine kinase activity and triggering two principal intracellular cascades: the PI3K/AKT pathway and the Ras/Raf/MEK/ERK (MAPK) pathway. These signalling routes regulate cytoskeletal organization and mediate INS function in cellular metabolism and growth responses [2].

Dysregulation of INS signalling—whether due to excess or deficiency—is a major initiating factor and/or contributor to metabolic disorders such as obesity, type 2 diabetes, dyslipidemia, atherosclerosis, and hypertension [3]. Numerous studies of INS-related disorders have focused on adipose (visceral) tissue, kidney, liver, and skeletal muscles, which are the main targets of the hormone [4]. INS also acts in regulatory processes in other districts, like the gastrointestinal (GI) tract. Intestinal epithelial INS signalling is involved in normal growth and intestinal development and modulates tight junctions and intestinal barrier permeability via protein synthesis and the expression of cytoskeleton structural elements [5,6,7]. As a modulator of glucose homeostasis, INS inhibits apical translocation of GLUT2 in enterocytes, thereby limiting glucose uptake from the intestinal lumen [8], and influences intestinal lipoprotein metabolism [9].

INS dysregulation, as well as other metabolic alterations, can intensely influence the whole GI system, including the oesophagus, stomach, upper and lower intestine, and gallbladder [10]. In particular, low-grade chronic systemic inflammation associated with derangement of INS homeostasis alters intestinal barrier morpho-functional features, leading to the impairment of both immunological and metabolic functions [11]. The association between barrier permeability and metabolic disorders is well described in animal models of obesity [12,13], as well as many clinical studies demonstrating the tight junction restructuring and mucosal barrier regulations in patients with metabolic disorders [14,15].

The occurrence of a leaky/hyper-permeable gut in INS-associated diseases is a factor that predisposes the translocation of microbiota-derived products to the bloodstream, provoking inflammatory activation [16,17]. In this regard, increases in LPS blood levels have been found and described in obese rodents and humans [18,19]; this evidence is directly related to the increase in intestinal permeability derived from the decreased expression of zonula occludens-1, claudin, occludin, and other proteins of the tight junction complex [20,21].

Imbalanced immune response induced by INS dysregulation is also characterized by the production of cytokines, which compromise INS signalling at multiple levels. Cytokines induce suppressors of cytokine signalling (SOCS), inhibiting the INS receptor’s function by preventing IRS-1/2 tyrosine phosphorylation, thus altering the proper signalling. Equally, pro-inflammatory pathways activated by cytokines, such as NF-κB and IKK, induce serine phosphorylation of IRS proteins, which limits the activation of downstream pathways [22]. Overall, the signalling pathways activated by cytokines lead to a feed-forward amplification, as demonstrated by increased levels of many circulating cytokines in obesity or diabetes [23]. The cytokine-induced alteration of INS signalling is studied specifically in INS target tissues. In adipose tissue, TNF-α alters IRS signalling by aberrant phosphorylation of IRS-1 serine [24]. Moreover, in adipocytes, a potent pro-inflammatory driver such as IL-1β decreases INS-driven glucose transport by inducing a reduction in IRS-1 mRNA transcription, which is dependent on ERK pathway activation. This weakening of INS signalling worsens the lipid buffering capacity in adipose cells, with consequent accumulation of lipids in muscle and liver [25]. Finally, IFN-γ specifically acts on skeletal muscle cells as a direct target to downregulate the INS receptor and enforce compensatory hyperinsulinemia to boost CD8^+^ T cells [26]. Beyond that, very little is known about the role of cytokines in INS signalling in the intestine; nevertheless, metabolic diseases are known to be associated with significant changes to the immune component in the intestine, which results in an aberrant inflammatory environment [27]. How and how much the inflammatory microenvironment hinders the metabolic function of INS in enterocytes remains unclear.

In this study, we describe the INS signalling pathways in differentiated enterocyte-like Caco-2 monolayers challenged by two pro-inflammatory mediators, i.e., IL-1β and IFN-γ, with the aim of identifying differential molecular and morpho-functional variations in association with INS-dependent responses in a model mimicking the inflamed intestinal epithelium in vitro.

## 2. Materials and Methods

### 2.1. Reagents and Materials

All chemicals, reagents, and kits were purchased at cell culture/molecular biology grade. Fetal bovine serum (FBS), Dulbecco’s phosphate-buffered saline (D-PBS), Eagle’s minimum essential medium (MEM), penicillin/streptomycin solutions, trypsin, L-glutamine, and non-essential amino acids were purchased from Corning-Fisher Scientific (Rodano, MI, Italy). The reagents 4′,6-diamidino-2-phenylindole (DAPI; Cat.: 28718-90-3), Triton X-100, paraformaldehyde (PFA; Cat. 30525-89-4), Phalloidin TRITC-conjugated (Cat.: P1951), and recombinant human insulin (91077C) were obtained from Sigma-Aldrich (Milano, Italy). Recombinant human interferon gamma (IFN-γ) and interleukin 1-beta (IL-1β) were supplied by Thermo Fisher Scientific (Monza, Italy).

### 2.2. Cell Culture and Treatments

Human epithelial Caco-2 cells (ATCC n. HTB-37™)were cultured according to previously adopted standard ATCC protocols [28]. For the experiments, Caco-2 cells were used at 21 days post seeding (dps) after continuous growth in standard culture conditions, to obtain spontaneously differentiated (“enterocyte-like”) epithelial monolayers according to consolidated differentiation protocols [28,29]. For the treatment assays, 0.5 × 10^5^ Caco-2 cells per well were seeded in 12-well plates (Corning-Fisher Scientific) and then cultured for 21 dps. At 21 dps, differentiated Caco-2 monolayers were exposed to different treatments with insulin (INS), IL-1β, and IFN-γ according to the following experimental design:

- INS: Cells were treated with 6 nM (1 UI/mL) insulin for 6 or 24 h;
- (IL-1β)INS: Cells were primed with 20 ng/mL IL-1β for 24 h. After stimulation the medium with IL-1β was removed and cells were washed twice with fresh medium. Soon after, cells were treated with 6 nM insulin for 6 or 24 h;
- (IFN-γ)INS: Cells were primed with 10 ng/mL IFN-γ for 24 h. Then, the medium containing IFN-γ was removed, and cells were washed twice with fresh medium. Immediately after, cells were treated with 6 nM insulin for 6 or 24 h.

### 2.3. Fluorescence Imaging of Cytoskeleton/Nuclei

A total of 0.5 × 10^5^ cells per well were seeded and grown on autoclaved, UV-sterilized glass coverslips deposited on the bottom of 12-well plate wells. After treatments, cells were washed with D-PBS (3 times) and fixed with 4% (*w*/*v*) PFA in D-PBS for 60 min at room temperature. Fixed samples were washed again with D-PBS (3 times) and then permeabilized for 20 min with 0.1% (*w*/*v*) Triton X-100/D-PBS (room temperature). For actin staining, cells were incubated (20 min) with 1 μg/mL phalloidin-TRITC in the dark and washed with D-PBS. Then, cell nuclei were counterstained by 5-min incubation with DAPI (0.1 mg/mL in D-PBS, lex 340 nm, lem 488 nm). Coverslips were finally mounted on glass slides with 1:1 glycerol/D-PBS, and image acquisition was performed with a Nikon Eclipse 800 microscope equipped with the Nikon-NIS-elements-D package suite software Version 3.07 (Nikon Europe, Amstelveen, The Netherlands).

### 2.4. Extraction of Total RNA and Proteins

RNA/protein extractions from cells were performed with the All-Prep DNA/RNA/Protein mini kit (Qiagen, Hilden, Germany) according to the manufacturer’s instructions. Briefly, cells in monolayer were double washed with D-PBS and then lysed with the kit lysis buffer by scraping. RNA concentrations were calculated by NanoDrop ND-2000 Spectrophotometer (Nanodrop Technologies, Wilmington, DE, USA), and RNA samples were qualitatively tested by electrophoresis on 1% (*w*/*v*) agarose gels. Concentrations of protein extracts were assessed by using the Bio-Rad Protein Assay kit (Bio-Rad, Segrate, MI, Italy), according to the manufacturer’s protocol [28].

### 2.5. Primer Design and Realt Time PCR (qPCR) Assays

For selecting oligonucleotide sequences to be used in real-time PCR (qPCR) gene expression analyses, primer design was performed by using mRNA reference sequences derived from the GenBank database (https://www.ncbi.nlm.nih.gov/gene, accessed on 29 October 2022). Gene-specific forward and reverse primer sequences were selected on different exons (intron-spanning) to avoid amplification of genomic DNA, and then the AmplifX software version 2.0.7 was used to test and check optimal amplification features of each selected primer pair. Details of the oligonucleotide sequences are reported in Table 1. Reverse transcription was run on 500 ng of total RNA, using 5x PrimeScript RT Master Mix (Takara Bio from Diatech Lab Line Srl, Jesi, AN, Italy) according to the manufacturer’s instructions. Before qPCR assays, primer pairs were tested for efficiency according to Schmittgen and Livak [30]. qPCR experiments were performed using the iTaq Universal SYBR Green Supermix (Bio-Rad) with a CFX96 Touch™ Real-Time PCR System (Bio-Rad). Relative quantification of mRNA expression was performed by analyzing the threshold values (C_T_) with the 2^−ΔCT^ method; qPCR results were expressed in the form of 2^−ΔCT^ values, as proportional to the amount of the specific mRNA target [30]. For each target gene and internal (housekeeping) control, 2^−ΔCT^ values were obtained from 2 different qPCR rounds for each of 6 biological replicates. Statistical analysis was performed after the 2^−ΔCT^ conversion [30].

### 2.6. Western Blotting

Electrophoresis of protein extracts was run on 12% Mini-PROTEAN TGX Stain-Free Protein Gels (Bio-Rad). Then, protein transfer to polyvinylidene fluoride (PVDF) membranes was performed. Subsequently, membranes were blocked for 5 min in EveryBlot Blocking Buffer (Bio-Rad) and immunoblotted in EveryBlot Blocking Buffer with primary and secondary antibodies (see Table 2 for dilutions and incubation times). The immunoreactive bands were detected with the enhanced chemiluminescence method (ECL kit, Bio-Rad) and ChemiDoc^TM^ Imaging System (Bio-Rad). According to the manufacturer’s instructions, densitometric quantification was carried out by using the Image Lab software version 6.1 (Bio-Rad), through which the pixel intensity for each band was analyzed, and the protein expression results were normalized with respect to the total lane of the loaded samples. Precision Plus Protein All Blue Standards (Bio-Rad, cat. no. #161-0373) was used as the molecular weight marker.

### 2.7. Statistical Analysis

All data were expressed as means ± standard deviation (SD). Data means were derived from two independent experimental assays in two independent cell cultures; for each assay, three biological replicates were performed (n = 6). According to references and experimental conditions with the Caco-2 cell line, reproducibility was based on assessing the optimal cell seeding number as well as the cell number at the experimental end-points [31,32,33]. Statistical analysis by one-way ANOVA followed by Dunnett’s multiple comparison test was performed using GraphPad Prism 9.4.0 software; *p* values ≤ 0.05 were considered significantly different.

## 3. Results

### 3.1. Effects of IL-1β and IFN-γ on Insulin Signalling Pathways

**Figure 1:**
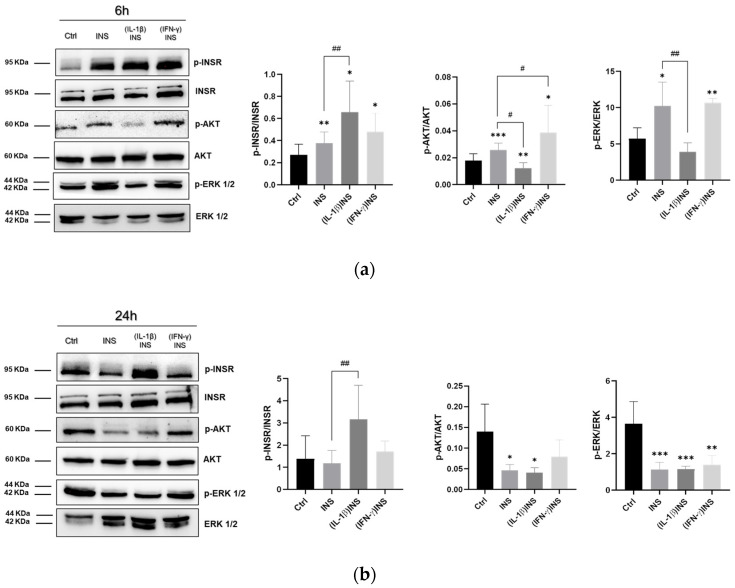
Expression analysis of key proteins of the insulin signalling pathways in intestinal epithelial monolayers exposed to inflammatory stimuli. Representative image of western blot assays performed using specific antibodies (see Section 2 for details) on protein extracts from differentiated Caco-2 monolayers exposed to insulin alone (INS) or pre-treated with cytokines [(IL-1β)INS; (IFN-γ)INS] before INS stimulation for 6 h (**a**) or 24 h (**b**). For each protein product, the molecular weight is shown on the left. The levels of phosphorylated INSR, AKT, and ERK 1/2 were corrected for the loaded amount of unphosphorylated INSR, AKT, and ERK 1/2, and represented as phosphorylated/unphosphorylated ratios in graphs. Data are represented as mean ± SD of three biological replicates from each of two independent experimental assays (n = 6) and then expressed as a percent with respect to the untreated control (Ctrl = 100%). Significant differences: *: vs. control; ^#^: vs. INS-treated cells; one-way ANOVA with Dunnett correction for multiple comparisons (*^/#^ *p* < 0.05; **^/##^ *p* < 0.01; *** *p* < 0.001).

First, we examined the activation of classical insulin signalling pathways in enterocytes. Differentiated Caco-2 monolayers were treated with 6 nM insulin (INS) for 6 or 24 h. After 6 h of stimulation, INS induced significant phosphorylation of the insulin receptor β subunit (INSR) as well as phosphorylation of downstream AKT and ERK 1/2 (Figure 1a), indicating activation of these pathways. Contrarily, after 24 h of INS exposure no changes in phosphorylated INSR were detected, while a parallel significant reduction of p-AKT and p-ERK 1/2 levels was observed compared to control cells (Figure 1b).

Next, we evaluated the INS signalling pathways in cells after having undergone stimuli mimicking intestinal inflammation. To this end, Caco-2 monolayers were pre-treated with IL-1β or IFN-γ for 24 h before INS administration. Both IL-1β and IFN-γ pre-treatment before 6 h of INS stimulation significantly enhanced phosphorylation of INSR (without affecting the amount of non-phosphorylated INSR protein; Figure 1a). Regarding the effects on downstream AKT and ERK 1/2 activations, the two cytokines regulated INS signalling through distinct mechanisms. Pretreatment with IL-1β prevented the INS-induced increase in AKT phosphorylation (in fact, p-AKT levels were found to be even lower than those in both the untreated control and INS-only treated cells); likewise, the INS-induced increaes in ERK 1/2 phosphorylation level was not detected in IL-1β pre-treated cells (Figure 1a). For both unphosphorylated AKT and ERK 1/2 proteins, pre-treatment with IL-1β did not affect their amount compared to untreated controls. On the other hand, pretreatment with IFN-γ significantly increased INS-induced phosphorylation of AKT compared to both untreated controls and cells treated with INS alone, while it induced a significant increase in p-ERK 1/2 levels respect to untreated controls only (Figure 1a). No significant changes in the quantity of unphosphorylated AKT and ERK 1/2 proteins were reported after IFN-γ pretreatments.

Then, AKT and ERK 1/2 levels were analyzed after prolonged exposure to INS (24 h). After pre-treatment with both cytokines, reductions in p-AKT and p-ERK 1/2 levels were detected compared to the untreated control monolayers (Figure 1b).

### 3.2. Insulin Induces Actin Cytoskeleton Remodelling in Inflammatory Conditions

**Figure 2:**
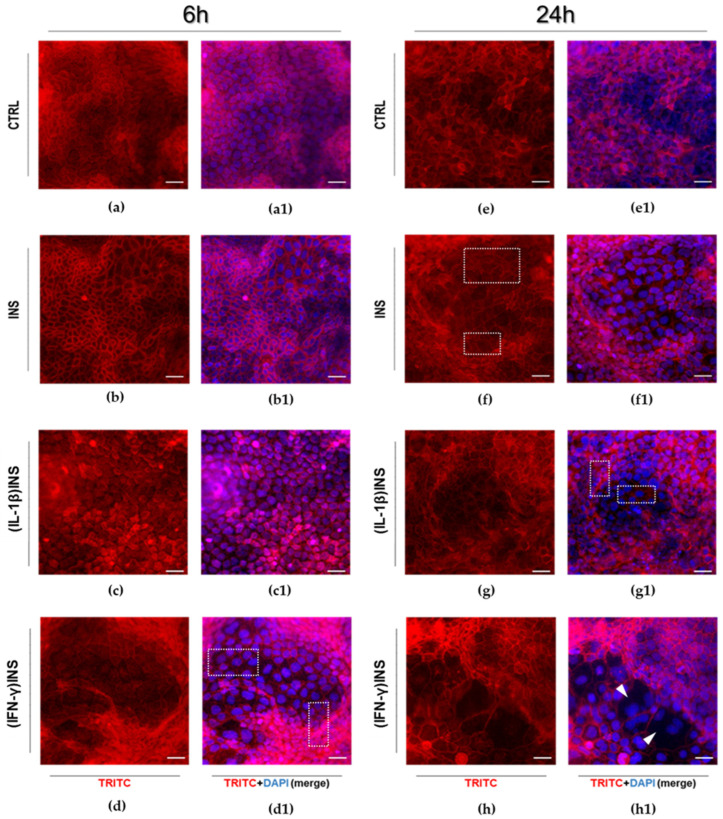
Morphological analysis after insulin stimulation (for 6 or 24 h) of differentiated Caco-2 monolayers exposed to inflammatory stimuli (IL-1β, IFN-γ). (**a**–**h**): Phalloidin-TRITC fluorescence alone; (**a1**–**h1**): merged images of phalloidin-TRITC (red) cytoskeleton staining and DAPI (blue) nuclear staining. White rectangles indicate areas in which the cytoskeletal actin elements are heterogeneously marked, with evident irregular cell-cell contact structures induced by the experimental treatments. The arrowheads show loss of regular cell-cell contact areas. Scale bar: 100 μm; 20× magnification.

We analysed the cellular model by investigating qualitative differences (untreated vs. INS-treated) and morpho-functional variations of the actin cytoskeleton elements. In the absence of specific treatments (Ctrl), Caco-2 mature (enterocyte-like) monolayers showed cytoskeletal homogeneity with highly regular mosaic-like organization of the differentiated cells, with detectable cell-cell contact/adhesion structures and physiological tightness (Figure 2a,e). In contrast, INS treatment induced changes in the actin cytoskeleton depending on time. With respect to untreated monolayers, in 6 h INS-treated monolayers (Figure 2b) intensification of the peri-junctional actin rings was detectable, while 24 h INS stimulation induced higher alteration of the F-actin fibres’ organization, i.e., more frayed and thickened actin structures appeared, with increased presence of cell-cell membrane infolding (Figure 2f, see representative areas in white rectangles and Supplementary Material File S1 for higher magnification).

Furthermore, the differentiated monolayers were primed for 24 h with IL-1β or IFN-γ and only after that were stimulated for 6 or 24 h with INS. Cells primed with IL-1β and next treated with INS for 6 h showed no specific alterations of the cytoskeletal structure compared to untreated control cells (Figure 2c); contrarily, after 24 h of INS stimulation, cells appeared different in shape and size compared to untreated controls, with cytoskeletal actin elements heterogeneously marked (thickened or disrupted, Figure 2g, see representative areas in white rectangles and Supplementary Material File S1 for higher magnification). IFN-γ administration induced the most significant effects on actin cytoskeleton organization in association with INS. Monolayers pre-treated with IFN-γ and then stimulated with INS for 6 h showed partial depolymerization of actin filaments in cell-cell contact areas with the loss of homogeneity in cell shape and size compared to untreated control cells (Figure 2d; see representative areas in white rectangles and Supplementary Material File S1 for higher magnification). Prolonged exposure to INS (24 h) of the monolayer pre-treated with IFN-γ remarkably enhanced the effects previously described: The organization of adjacent cells was strongly altered, showing severe discontinuity in cell-cell borders (Figure 2h, white arrowheads). In contrast to the highly regular 2D architecture of untreated control monolayers, the mosaic-like organization underwent substantial alterations, with cells losing the regular polygonal geometry, becoming enlarged with partly roundish shape and variable size (Figure 2h).

**Figure 3:**
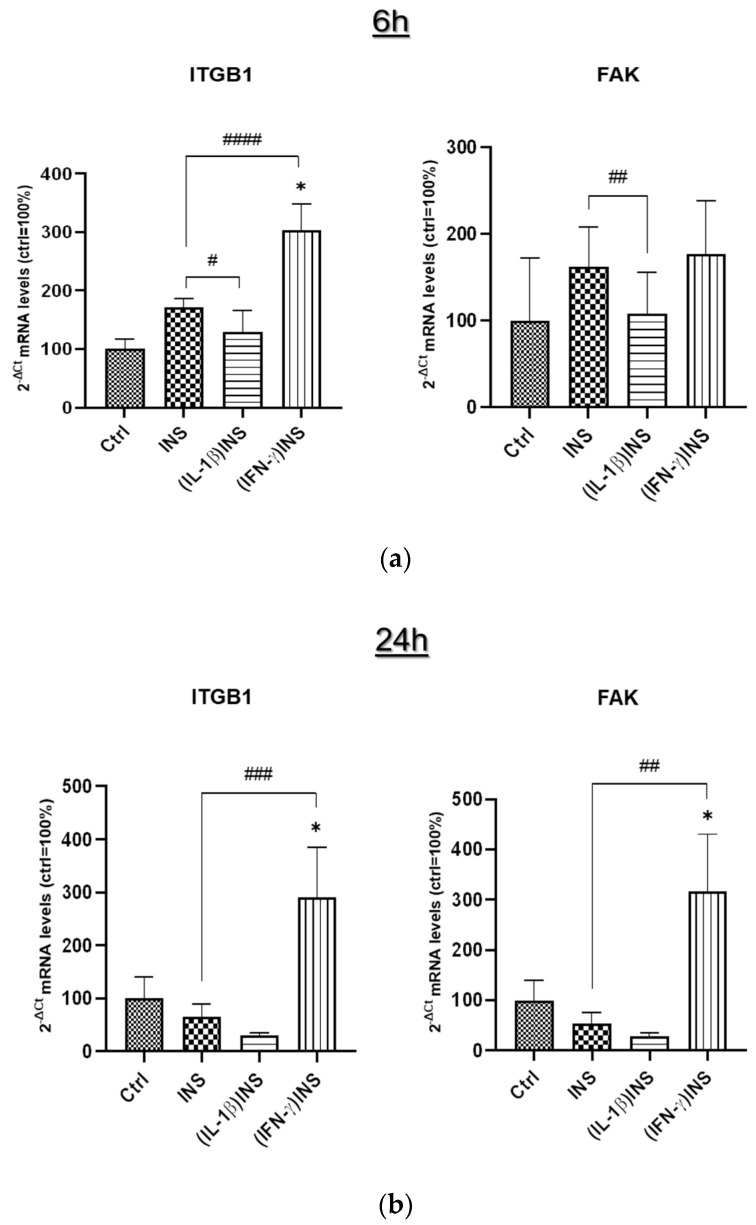
mRNA expression analysis by real-time PCR of cytoskeletal-related genes after insulin stimulation for (**a**) 6 h or (**b**) 24 h in differentiated Caco-2 monolayers exposed to inflammatory stimuli (IL-1β, IFN-γ). Amounts of target mRNAs (ITGB1 and FAK) were calculated as 2^−ΔCt^ mean values obtained from two rounds of real-time PCR assays for each of three independent biological replicates from each of two independent experimental assays (n = 6) and normalized with respect to the GAPDH gene. Then, values were expressed as percent ± SD with respect to the untreated control mean value (Ctrl = 100%). Significant differences: *: vs. control; ^#^: vs. INS-treated cells; one-way ANOVA with Dunnett correction for multiple comparisons (*^/#^ *p* < 0.05; ^##^ *p* < 0.01; ^###^ *p* < 0.001; ^####^ *p* < 0.0001).

In parallel, the mRNA expression analysis of genes related to cytoskeletal dynamics, i.e., integrin β1 (ITGB1) and focal adhesion kinase (FAK), has been performed to check the activation of morphological processes undergoing the experimental conditions. According to the morphological analysis of cytoskeletal actin, mRNA expression variations induced by INS have been shown to follow a general dependence on time (Figure 3). After 6 h of INS stimulation, FAK mRNA expression analysis showed no significant variations in all experimental conditions compared to untreated controls; nevertheless, its expression levels were found to be significantly lower in (IL-1β)INS monolayers compared to INS-treated cells (Figure 3a). The ITGB1 mRNA underwent a regulatory trend similar to FAK: Its expression was significantly reduced by pre-treatment with IL-1β compared to INS-treated cells, but, different from FAK, ITGB1 mRNA was significantly increased by IFN-γ pre-treatment compared to both untreated controls and INS-treated cells (Figure 3a). Interestingly, in cells exposed to INS for 24 h, the qPCR analysis showed an identical trend for both ITGB1 and FAK mRNAs, with the (IFN-γ)INS treatment inducing significant upregulation of both mRNAs compared to both untreated controls and INS-treated cells (Figure 3b); moreover, this upregulation was much more marked than that observed with only 6 h of INS stimulation.

## 4. Discussion

Recent scientific evidence is constantly showing that inflammation of GI epithelia may represent an early event that precedes and predisposes to insulin-related disorders [3]. This work is focused on the analysis of insulin signaling pathways in an in vitro model of GI tract inflammation, with specific attention to the differential effects induced by the two pro-inflammatory cytokines IL-1β and IFN-γ, to check for alterations related to the INS metabolic pathway as a function of intestinal inflammation. In this context, the human Caco-2 cells proved to be optimal, based on their well-known propensity for spontaneous differentiation towards mature epithelial monolayers, showing morpho-functional properties of small intestine enterocytes [28,29]. In this work, the 6 nM INS concentration was chosen according to the scientific literature dealing with the use of INS in in vitro cell cultures applied to studies on INS concentration in human clinical issues; in particular, 6 nM concentration corresponds to 1 U/mL [34,35,36]. While studies with INS-based treatments on enterocytes are very scarce, there are studies that deal with the in vitro induction of insulin resistance, for example, in adipocytes or muscle cells. In these studies, the INS concentrations vary in a wide range, from a few units to >100 nmol/L [37]; since our “ab initio” investigation in enterocytes primarily aimed to identify molecular and/or morphological adaptations to the presence of INS, we have chosen a mild concentration, albeit administered for two substantially different times (6 and 24 h).

Here, the regulation of INS signaling in intestinal epithelial cells was investigated in relation to the activation (phosphorylation) state of INSR, AKT, and ERK 1/2 in differentiated Caco-2 monolayers. Six hours of INS stimulation increased levels of all the phosphorylated proteins. These results are associated with INS-specific effects on the monolayer’s cytoskeletal architecture. INS induced the intensification of peri-junctional actin rings, in agreement with its known effects of the hormone on cell morphology [1]. In this respect, genes related to the cytoskeletal dynamics, i.e., *FAK* (focal adhesion) and *ITGB1* (cytoskeleton/ECM cross-talk), respond to INS stimulation according to the monolayer remodeling. These evidence highlights that INS-induced remodeling dynamics with rearrangement of specific actin structures is associated with the effective stimulating action of INS on enterocytes, according to the significant activation of INS-related signaling pathways.

The analysis of cytoskeleton dynamics and activation of INS signaling after 24 h of insulin stimulation showed that specific alterations are time-dependent. It is known that constantly elevated levels of INS may cause imbalance of cellular homeostasis and organ functionality as well [38]. When we exposed differentiated Caco-2 cells to INS stimulation for 24 h, we observed a deficit in INSR receptor phosphorylation compared to that observed after a 6 h stimulation, which was shown to occur in the absence of significant changes in the amount of INSR (Figure 2). Notably, we detected the downregulating dysregulation of the effectors AKT and ERK 1/2. It is known that long-term INS exposure leads to lesser INS sensitivity. In order to maintain homeostasis, several mechanisms are routinely activated to prevent an increase in INS signaling under conditions of INS excess, such as decreased INSR kinase activity and reduced downstream signal transduction [39]. Thus, our results showed a physiological response of enterocytes to prolonged INS exposure (24 h), in accordance with other studies in isolated adipocytes and liver cells [40,41]. Moreover, Caco-2 cells subjected to 24 h INS exposure showed more significant alterations in cytoskeletal actin with an increase in membrane ruffling structures compared to cells stimulated for 6 h (Figure 2b–f), as has been previously described for 3T3-L1 adipocytes and muscle cells treated with physiological doses of INS for a long time [42,43]. This suggests that “chronic” exposure to INS significantly affects enterocytes in monolayers, both at the INS signaling transduction level and at the actin structure level.

Alteration of INS-dependent pathways’ activation may contribute to a state of reduced INS sensitivity. Among the changes that occur in association with insulin resistance, there are growing levels of certain inflammatory cytokines (i.e., IL-1β, IL-6), which contribute greatly to a chronic low-grade systemic inflammation that is a critical factor in insulin resistance and metabolic dysfunction, especially in type 2 diabetes [22,25]. As described by our results, the inflammatory state induced by pre-treatment of enterocyte-like monolayers with IL-1β or IFN-γ effectively altered INS signaling in cells. Pro-inflammatory cytokines compromise INS signaling by multiple mechanisms [3]. TNF-α, a well-known cytokine involved in the development of metabolic dysfunction, can indirectly act to regulate energy metabolism, since its pro-inflammatory effects impact the production of adipokines and cytokines [44]. Recently, it was demonstrated that TNF-α can impair INSR receptor signaling by stimulating Ca^2+^ release from the endoplasmic reticulum, thus increasing intracellular Ca^2+^ levels and activating Ca^2+^/calmodulin-dependent protein kinase II [45]. IL-1β, a major pro-inflammatory cytokine, is critically involved in INS signaling alterations; in obese individuals, high IL-1β and IL-6 circulating levels represent an important independent risk for developing type 2 diabetes [46]. In addition, in vitro studies have described the pathogenic impact of IL-1β on INS pathways and the potential enhancement of INS sensitivity by IL-1β signaling inhibition [47]. As we have experimentally assessed, pre-treatment with IL-1β followed by 6 h of INS stimulation induced INSR activation on one hand, but diminished INS-induced phosphorylation of AKT and ERK 1/2 on the other, compared to cells treated with INS alone. This downregulation of AKT and MAPK-dependent pathways may contribute to a state of reduced INS sensitivity in differentiated Caco-2 cells in monolayer, strongly indicating that dysregulation of INS signaling also occurs in intestinal epithelial cells with all its pathogenic potential. Notably, we found that IL-1β pre-treatment before INS administration did not appear to produce specific evidence of morpho-functional alterations of the actin cytoskeleton. The functional effects of signaling events on cytoskeleton and ECM are well established. In particular, AKT and MAP kinase (ERK 1/2) pathways are largely intertwined with the cross-talk routes for cytoskeleton dependent control [48]. Detailing our findings, the observed downregulation of these pathways induced by IL-1β in enterocyte-like cells resulted in no specific alterations of the cytoskeletal structure and no variations in ITGB1 and FAK mRNA levels compared to untreated cells. Particularly, these findings agree with previous data from gastric cancer cells, in which suppression of PI3K/AKT by the PI3K inhibitor LY294002 or silencing AKT leads to decreased Wnt5-induced GSK-3 phosphorylation, which further causes reduced RhoA-dependent cell migration and actin remodeling [49].

The analysis of the effects of IFN-γ pre-treatment on INS signaling in enterocyte-like Caco-2 monolayers showed that its inflammatory impact is different from that of IL-1β. Indeed, the synergistic effect of IFN-γ and INS predominantly induced activation of AKT and ERK 1/2 in association with the most significant alterations of the monolayer’s cytoskeletal dynamics, as also confirmed by the analysis of FAK and ITGB1 mRNA expression. Interferons are a family of cytokines that play key roles not only in inflammation and immune contexts but are also related to metabolic diseases [50]. IFN-γ causes insulin resistance in skeletal muscle [26] and stimulates phenotypic changes in human adipocytes impaired by INS action [51]. In our Caco-2 monolayers primed with IFN-γ, 6 h of INS treatment induced an increase in p-AKT and p-ERK 1/2. Activation of both AKT and ERK 1/2 pathways appears to be involved in the alteration of the actin cytoskeleton in different cell lines; in fact, their inhibition is sufficient to restore the cytoskeletal actin organization [52,53,54,55]. Our data in Caco-2 monolayers showed that overactivation of these two pathways in the (IFN-γ)INS condition occurs concomitantly with actin cytoskeleton disorganization (i.e., derangement of cell-cell contact areas and inhomogeneity of cell shape/size). Remodeling associated with activation of AKT and MAP kinase pathways is more significant when INS treatment is preceded by IFN-γ stimulation than by IL-1β. Indeed, it is well known that IFN-γ disrupts the barrier function of tight junctions (TJ) across cultured epithelial monolayers [56]; moreover, in differentiated Caco-2 monolayers, it has been described that exposure to IFN-γ rapidly modifies the localization of TJ proteins (ZO-1, claudin-5, and occludin) and elicits depolymerization of peri-junctional cytoskeletal F-actin patterns [57]. It is therefore conceivable that, in our hands, IFN-γ and INS provide the cue for an evident reorganization of the actin cytoskeleton.

Clearly, longer-term INS exposure (24 h) worsens F-actin organization in inflamed monolayers. Indeed, IL-1β- or IFN-γ-primed Caco-2 cells stimulated with INS for 24 h showed an overall significant cytoskeletal alteration/remodeling compared to cells exposed for a shorter time (6 h). In both (IL-1β)INS and (IFN-γ)INS conditions, cytoskeletal actin elements appeared thickened and damaged, with strong derangement of the monolayer’s mosaic-like organization. In particular, (IFN-γ)INS induced the most significant alterations of cytoskeletal dynamics, also associated with dysregulation of ITGB1 and FAK transcriptional expression, compared to INS-treated cells. Notably, *ITGB1* and *FAK* genes are interrelated not only with actin cytoskeleton rearrangements but also with intracellular signaling, cytokine activation, and release; therefore, they play an important role in cell proliferation, polarity, shape, and differentiation, as well as in all processes critical to inflammation [58,59]. In agreement with our results, it has already been observed that IFN-γ stimulates the expression of integrins in gastric cancer cells [60]. Moreover, the actin remodeling observed in inflamed monolayers treated long-term with INS is associated with the downregulation of the insulin signaling described for cells treated with 24 h of INS alone. In other words, data indicate that prolonged INS exposure alters its signaling and impairs cytoskeleton/ECM cross-talk in enterocyte monolayers regardless of the proinflammatory experimental conditions.

In the comprehensive framework of inflammation onsets and states along the gastrointestinal districts, it is worth noticing that altered activation of insulin pathways may be bidirectionally intertwined (as cause or effect) with the complex pattern of the entire suite of gut hormones (e.g., glucagon-like peptide-1 and -2, glucose-dependent insulinotropic polypeptide, peptide YY, cholecystokinin, apolipoprotein A4), as reported for the prototypical clinical context of intestinal inflammation, i.e., IBD [61]; the same applies to the more clinically relevant dysfunctions affecting metabolic health [62,63]. Consequently, although our data are limited by the need to necessarily (quantitatively) deepen the morphological evidence preliminarily provided, our direct evidence of certain differential actions of different cytokines may serve to better identify some key relationships between some hormonal regulations in inflammation, with particular reference to the main intestinal interface, i.e., the enterocytic monolayer.

## 5. Conclusions

Overall, our results suggest a picture of responsiveness of the intestinal epithelial monolayer to insulin in the inflammatory environment, to be investigated also in the context of conditions driven by insulin resistance and affecting functions of the enterocyte monolayer at different levels. Intriguingly, the differential impact of different cytokines/inflammatory mediators has been described for the first time as a precondition that can influence the impact of insulin on the intestinal epithelium, taking on the meaning of a prerequisite capable of piloting the transition from physiological function to physiopathological onset in an inflamed gastrointestinal context, both in a setting of insulin resistance and in its absence.

## Figures and Tables

**Table 1:** Primer sequences for qPCR expression analysis. For the *ITGB1*, *FAK*, and *GAPDH* genes, the NCBI accession numbers of the mRNA sequences (RefSeq mRNA) used for primer design are reported. For each sense and antisense primer, the 5′-3′ nucleotide sequence and melting temperature (Tm) are reported. The expected amplicon length (PCR size) is also indicated in base pairs (bp).

GENE	RefSeq mRNA	Sense primer 5′-3′ (Tm)	Antisense Primer 5′-3′ (Tm)	PCR Size (bp)
*ITGB1*	NM_002211.3	CAAATGCCAAATCATGTGGA (55 °C)	TTCTCTGCTGTTCCTTTGCT (55 °C)	225
*FAK*	L13616.1	ATTAAATGGATGGCTCCA (55 °C)	CTCCCACATACACACACC (58 °C)	121
*GAPDH*	NM_001256799.3	AAACCTGCCAAGTATGATGA (51 °C)	TACTCCTTGGAGGCCATGT (54 °C)	217

**Table 2:** Features of the antibodies for Western blotting (WB) assays. For each primary and secondary antibody, the commercial product ID and a summary of the protocol steps adopted for WB analysis are reported.

Antibody	Product ID	WB	
		Dilution	Incubation Time
Phospho-INSR (Tyr 1150/1151) (Santa Cruz from DBA, Segrate, MI, Italy)	sc81500	1:1000	Overnight, 4 °C
INSR (GeneTex from Prodotti Gianni, Milano, Italy)	GTX101136	1:1000	Overnight, 4 °C
Phospho-p44/42 MAPK (Erk 1/2) (Thr 202/Tyr204) (Cell Signaling from DBA, Segrate, MI, Italy)	#4377	1:1000	Overnight, 4 °C
p44/42 MAPK (Erk 1/2) (Cell Signaling)	#9102	1:1000	Overnight, 4 °C
Phospho-AKT (Ser 473) (Cell Signaling)	#9271	1:500	Overnight, 4 °C
AKT (Cell Signaling)	#9272	1:1000	Overnight, 4 °C
Anti-mouse IgG, HRP-conjugate (Sigma-Aldrich)	AP308P	1:5000	1 h, room temperature
Anti-rabbit IgG, HRP-linked (Cell Signaling)	#7074	1:1000	1 h, room temperature

## Data Availability

The original contributions presented in this study are included in the article/Supplementary Materials. Further inquiries can be directed to the corresponding author(s).

## Supplementary Materials

The following supporting information can be downloaded at: https://www.mdpi.com/article/10.3390/cells14171358/s1, File S1: Graphical details and magnifications.
