# Supplementary material for: Morpho-Functional Responsiveness of Caco-2 Enterocyte-like Monolayers to Insulin in a Pro-Inflammatory Environment
Source: Cells. 2025 Aug 31;14(17):1358. doi: 10.3390/cells14171358 (PMC12427942; doi:10.3390/cells14171358)

cells

MDPI

Article

Morpho-functional Responsiveness of Caco-2 Enterocyte-like Monolayers to Insulin in a Proinflammatory Environment

Aurora Mazzei [1], Marina Damato [2], Ilenia Iaia [3], Michele Maffia [2], Roberta Schiavone [1], Tiziano Verri [1], Amilcare Barca [4]*

[1] Laboratory of Applied Physiology, Department of Biological and Environmental Sciences and Technologies, University of Salento, Lecce, 73100, Italy; aurora.mazzei@unisalento.it (A.M.); roberta.schiavone@unisalento.it (R. S.); tiziano.verri@unisalento.it (T.V.)
[2] Laboratory of Human Physiology, Department of Experimental Medicine, University of Salento, Lecce, 73100, Italy; marina.damato@unisalento.it (M.D.); michele.maffia@unisalento.it (M.M.)
[3] Institute of Clinical Physiology, National Research Council IFC-CNR, Lecce, 73100, Italy; ileniaiaia@cnr.it
[4] Laboratory of Applied Physiology, Department of Experimental Medicine, University of Salento, Lecce, 73100, Italy
* Correspondence: amilcare.barca@unisalento.it

SUPPLEMENTARY MATERIAL S1: Graphical details and magnifications

Graphical details as reported in the text describing the results represented in Fig. 2 from section 3.2. Insulin induces actin cytoskeleton remodelling in inflammatory conditions

Figure 2f – *Morphological analysis after insulin stimulation for 24 h of differentiated Caco-2 monolayers*. Details in representative areas (see white rectangles as desrcribed in Fig. 2). With respect to 6 h INS treatment, after 24 h INS stimulation induces higher alteration of the F actin fibres' organization which displayed more frayed and thickened structures, with increased presence of cell-cell membrane infolding (see yellow asterisks in picture). The cytoskeletal actin elements are heterogeneously marked, with evident irregular cell-cell contact structures. (**f**): phalloidin-TRITC fluorescence alone; (**f1**): merged images of phalloidin-TRITC (red) cytoskeleton staining and DAPI (blue) nuclear staining. 40X magnification.

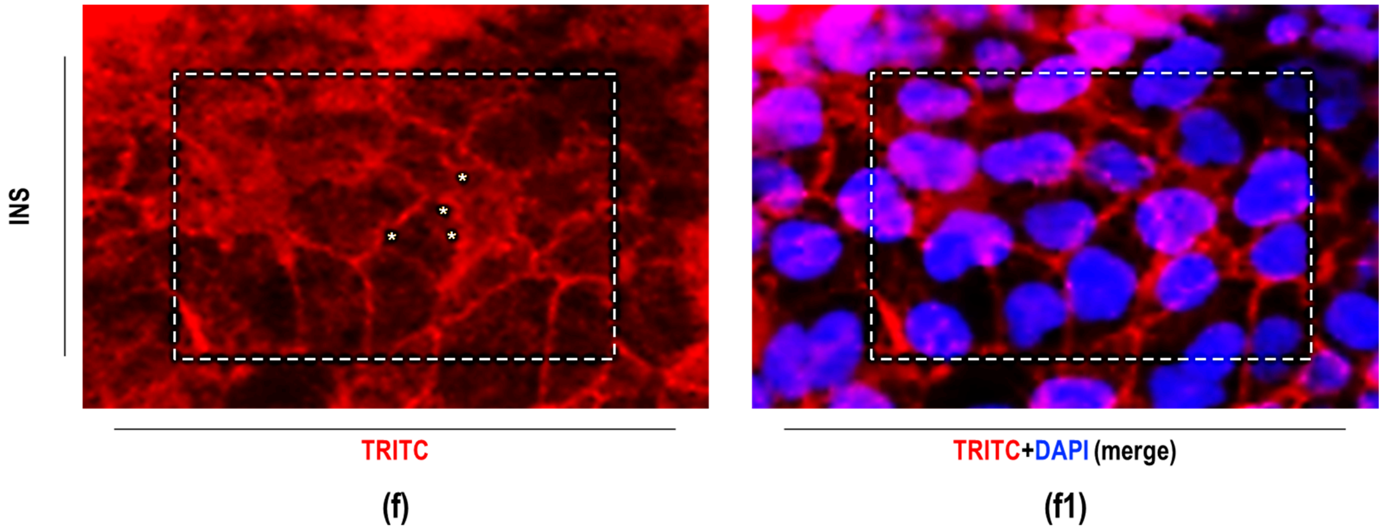

Figure 2g - *Morphological analysis of differentiated Caco-2 monolayers primed with IL-1β and next stimulated with INS for 24 h.* Details in representative areas (see white rectangles as desrcribed in Fig. 2). After 24 h of INS stimulation, IL-1β-primed cells appeared different in shape and size respect to controls, with high heterogeneity of the fluorescent marking of cytoskeletal actin elements (see different areas with thickened vs. disrupted actin cytoskeleton in figure). (**g**): phalloidin-TRITC fluorescence alone; (**g1**): merged images of phalloidin-TRITC (red) cytoskeleton staining and DAPI (blue) nuclear staining. 40X magnification.

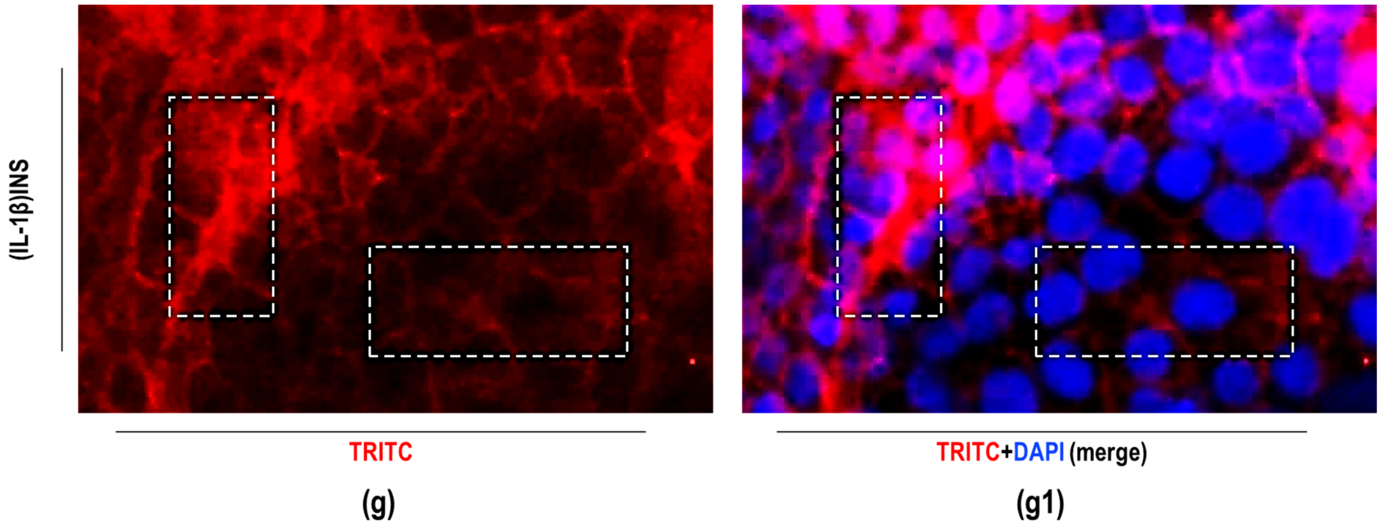

Figure 2d - *Morphological analysis of differentiated Caco-2 monolayers primed with IFN-γ and next stimulated with INS for 6 h.* Details in representative areas (see white rectangles as desrcribed in Fig. 2). Monolayers pre-treated with IFN-γ and then stimulated with INS for 6 h show partial depolymerization of actin rings/filaments in cell-cell contact areas with the loss of homogeneity in cell shape and size compared to control cells. (**d**): phalloidin-TRITC fluorescence alone; (**d1**): merged images of phalloidin-TRITC (red) cytoskeleton staining and DAPI (blue) nuclear staining. 40X magnification.

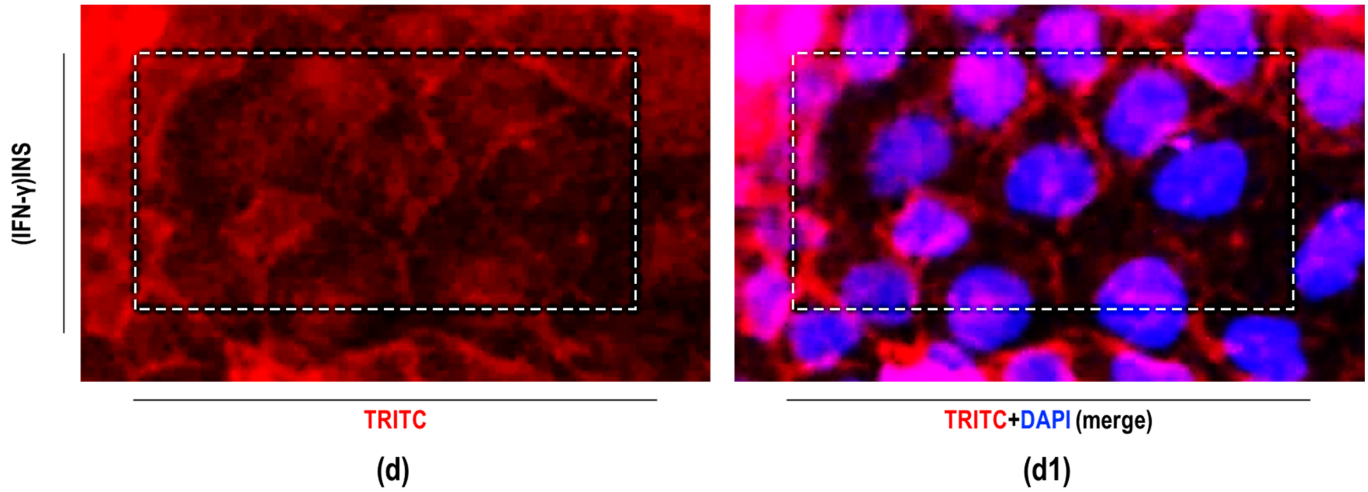

Supplement: Supplementary file 1 [file cells-14-01358-s001.zip › cells-3780454-supplementary.pdf]
